# MXene-Coated Liquid Metal Nanodroplet Aggregates

**DOI:** 10.1021/acs.langmuir.5c00173

**Published:** 2025-03-26

**Authors:** Mason Zadan, Yafeng Hu, Jeremiah Lipp, Michael Vinciguerra, Neal Lewis, Dylan Shah, Mohammad F. Islam, Dhriti Nepal, Matthew Grasinger, Kaushik Dayal, Christopher Tabor, Carmel Majidi

**Affiliations:** † Mechanical Engineering Department, Carnegie Mellon University, Pittsburgh, Pennsylvania 15213, United States; ‡ Materials and Manufacturing Directorate, Air Force Research Laboratory, Dayton, Ohio 45433, United States; § Materials Science and Engineering Department, Carnegie Mellon University, Pittsburgh, Pennsylvania 15213, United States; ∥ Arieca Inc., Pittsburgh, Pennsylvania 15208, United States; ⊥ Civil and Environmental Engineering Department, Carnegie Mellon University, Pittsburgh, Pennsylvania 15213, United States

## Abstract

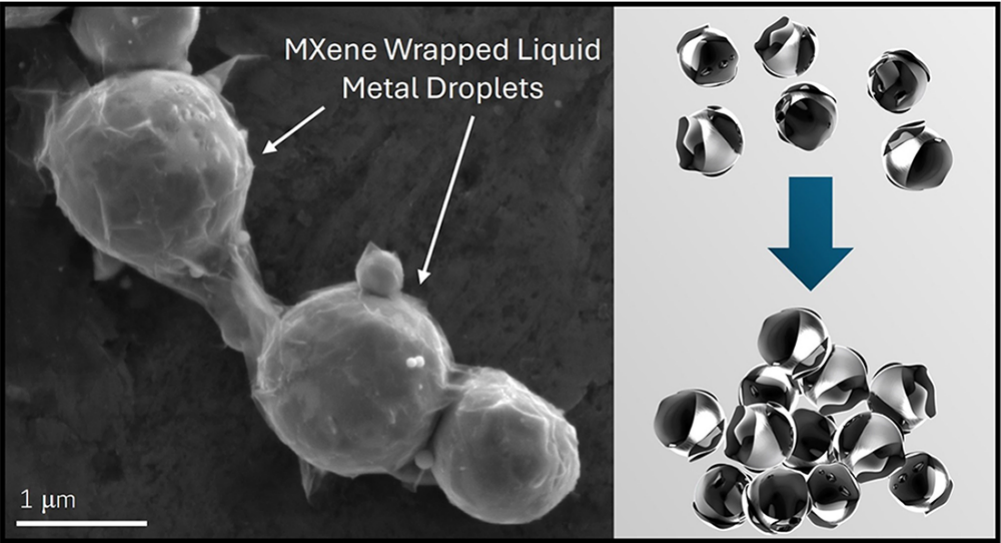

Combining droplets of liquid metal (LM) with nanomaterials often introduces synergistic thermal or electrical properties that are not found in the constituent materials alone. However, in these existing systems, LM droplets maintain a statistically uniform dispersion and are not capable of self-assembly or aggregation. These composites are limited by their need for high volume fractions of LM (>60 vol %) to achieve high thermal properties, introducing LM leaking as a drawback for thermal management and wearable electronic applications. In this work, we show that coating nanoscale droplets of eutectic gallium–indium (EGaIn) LM with small volume fractions of Ti_3_C_2_T_*x*_ MXenes (0.25 vol %) results in a unique LM morphology in which droplets self-assemble to form semisolid aggregates. This is accomplished by wrapping MXene sheets around individual LM droplets to create “sticky” particles that form self-assembled aggregates when mixed with a silicone oil. By introducing aggregation as a design parameter in soft LM composites, the thermal and electric resistance of the composite is shown to change dramatically. In contrast to silicone-based composites containing LM droplets or MXene nanosheets alone, these MXene-LM-silicone-based composites exhibit an exponential increase in thermal and electrical conductivity with decreasing interfacial thickness with significantly lower LM volume fractions (25 vol %) while avoiding LM rupture and bleed-out. This could enable more effective composites, reducing the amount of filler material required for thermal interface materials (TIM) and printed electronics.

## Introduction

Nanomaterial systems composed of liquid metal (LM) alloys such as eutectic gallium–indium (EGaIn) can be engineered to exhibit unique combinations of nanoscale morphologies, microstructures, and material properties. These systems are typically composed of dispersions of nano/microscale droplets (∼0.1–10 μm) of EGaIn that are suspended within a solvent or embedded in a polymer medium. When combined with other nanomaterials or microscale particles, these dispersions can exhibit a variety of emergent mechanical, thermal, and electrical properties that cannot be achieved with the individual constituent materials alone. This has included the introduction of rigid fillers such as Ag flakes into LM composites, which exhibited improved electrical performance through the formation of AgIn particles within the material.^1^ The introduction of Cu particles into EGaIn exhibited modest thermal conductivity improvements.^2^ Hu et al. showed that coating EGaIn with polydopamine improves dielectric performance.^3^ Separately, insulating graphene oxide coated EGaIn particles exhibited improved stability in extreme pH environments.^4^ Recently, core–shell polymer-LM composites have become an emerging material for electronics, injectable biomedicine solutions, and thermal management.^5,6^ In particular, surface-initiated atom transfer radical polymerization (ATRP), used to form and coat EGaIn particles, has shown improved thermal and optical properties along with a decrease in melting point through the suppression of crystallization.^7^

While the synergistic interplay between EGaIn nanodroplets and other nanomaterials leads to a wide variety of emergent properties, these existing material systems do not allow for direct control of the LM interactions or aggregation. In most cases, the LM droplets within a nanomaterial system form a statistically uniform dispersion and will not stick together or phase separate from the surrounding dispersion medium. These limitations lead to the requirement that LM composites must have exceedingly high LM volume fractions. Only at high volume fractions (>60 vol %) do these composites exhibit electrical conductivity or high thermal conductivities leading to the possibility of LM leakage. This problem could be addressed by developing LM droplets that can self-assemble into aggregates by introducing a “sticky” 2D coating that both conforms to the surface of the droplets and also promotes adhesion between droplets. MXenes are a promising candidate as a sticky coating, on account of their atomically thin form factor and high thermal and electrical conductivity, which could preserve the ability to maintain high conductivity between coated LM droplets within an aggregate. MXenes are a class of 2D conductive materials that include transition metal carbides, nitrides, and carbonitrides made of alternating intercalated metallic layers capped with a termination layer.^8,9^ These materials exhibit strong negative surface charges and high stability when exfoliated and introduced into polar aprotic solvents.^10^ The thermal and electrical performance of the MXene flakes Ti_3_C_2_T_*x*_ has been reported to be as high as 2.4 × 10^6^ S/m^11^ and 42.2 W/m/K^12,13^ for MXene films, respectively. Previous work has introduced this material for a wide variety of applications, including energy harvesting,^14^ energy storage,^15^ flexible electronics,^16^ and soft-matter composites.^17^ Recent work has also investigated LM-MXene amalgams to control malleability and printability for EM shielding^18^ along with decreasing corrosion and improved wettability of the LM.^19^

Recently, researchers have begun investigating the use of MXenes and LM to improve battery anode performance. For example, Zhang et al. distributed LM particles within assembled 3D MXene cells to make more robust anodes (489 mAh/g at 5 A/g).^20^ In another study, LM particles have been dispersed to connect MXene sheets to form MXene cages around Si microparticles,^21^ while Liu et al. uniformly distributed LM between MXene sheets to form porous meshes.^22^ Although promising for these specific applications, the literature demonstrates LM particles distributed within MXene networks and does not demonstrate an ability or process to form individually wrapped MXene-LM particles or investigate the emergent properties of the subsequent interparticle interactions that may occur when functionalized MXene-LM particles come in contact with eachother. More specifically, the influence of the MXene volume percentage on the performance of the LM composites remains to be shown. Lastly, we note that the thermal and electrical benefits of the MXene-LM interactions are currently under-studied, despite the high potential for such a system to create thermal interface materials (TIMs)^23^ with improved thermal and electrical performance, an area with applications in critical industries including semiconductors, electric vehicles, and power electronics.

**Figure 1:**
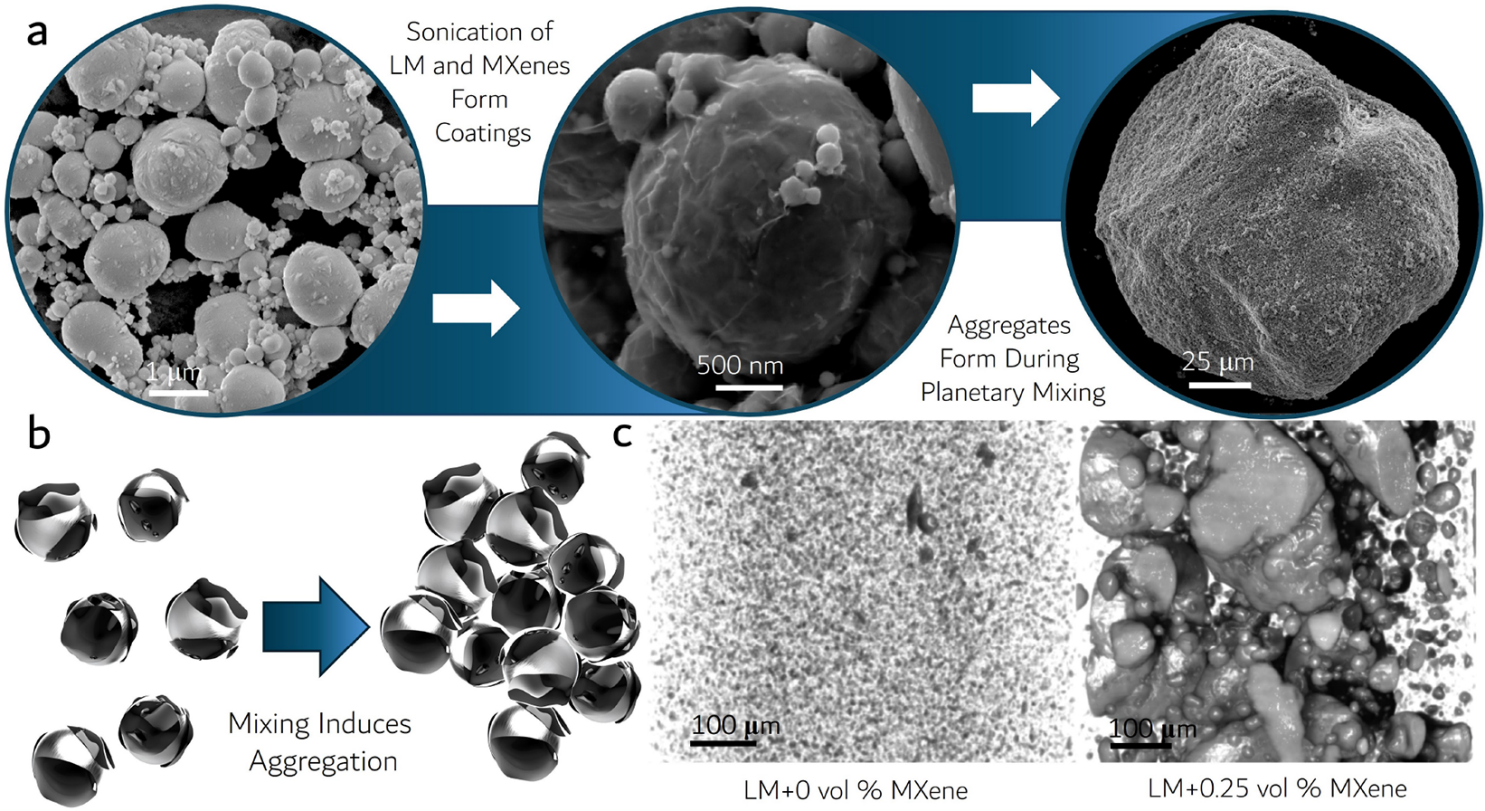
MXenes Coat LM During Synthesis: (a) Synthesis process of MXene-LM composites. Left: EGaIn nanoparticles shown after being broken up by probe sonication. Middle: SEM image of a representative high MXene content composite (LM+9.7 vol % MXene) after Ti_3_C_2_T_*x*_ MXene sheets were probe sonicated with the EGaIn nanodroplets creating a full coating of MXenes around the LM. Right: after solvent removal a silicone oil matrix was added to the composite and mixed, causing the functionalized MXene-LM composites particles to aggregate into large clusters as shown in the image of this LM+0.5 vol % MXene aggregate. (b) Graphic highlighting the fully MXene coated LM spheres and how these “sticky” particles aggregate into clusters after mixing. (c) Calibrated microCT reconstructions showing the influence of a small amount of MXenes on particle aggregation vs an LM control sample.

In this work, we investigate Ti_3_C_2_T_*x*_ MXene nanosheets as a coating and binder for nanoscale LM droplets, enabling the formation of self-assembled aggregates within a silicone-based dispersion medium. In particular, we demonstrate how small MXene volume fractions (e.g., ∼0.25 vol %) induce LM aggregation and have an outsized effect on composite morphology and performance. We demonstrate that even at low LM volume percentages (25%), the introduction of small amounts of MXenes (0.25 vol %), induces the normally electrically insulating LM droplets (∼10–400 nm diameter) to self-assemble into large aggregates (∼150 μm diameter) (Figure 1a). When embedded within a noncuring silicone-based matrix, the aggregated MXene-LM aggregates exhibit an exponential improvement in thermal conductivity and at low composite thicknesses transform the LM composite material from acting as an electrical insulator to a conductor. These aggregates demonstrate leak-free LM composites requiring a much lower LM volume to achieve comparative performance.

To synthesize this MXene-LM system, we introduce a solvent-based process that utilizes probe sonication to break up bulk EGaIn. This is followed by fully wrapping the nanoscale EGaIn droplets using probe sonication with exfoliated MXene sheets to form microclusters (Figure 1b). This MXene coating is shown to functionalize the surface, causing these LM particles to become “sticky” and capable of self-assembling into larger aggregates that are on the order of ∼150 μm in diameter. When suspended within a silicone oil, MXene-LM aggregates can function as a paste with enhanced thermal and electrical properties compared to those of LM-silicone suspensions that do not include MXene (Figure 1c). Because of the malleability of these soft composites, thermal and electrical conductivity of these materials is observed to increase nonlinearly as the interface distance decreases to below the nominal diameter of the MXene-LM aggregates within the composite.

## Experimental Section

### Liquid Metal Preparation

MXene-LM composites were prepared by using a solvent-assisted probe sonication process. First, an 80:20 DMSO:DI water solution was prepared. DMSO is a polar aprotic solvent that stabilizes, and when introduced with probe sonication, exfoliates MXene sheets and mitigates MXene aggregation and restacking during synthesis.^10^ This DMSO solution was degassed, removing oxygen to prevent the growth of additional oxide, which can inhibit the breakup of EGaIn droplets to the micron and submicron scale during probe sonication.^24^ Next, we alloyed gallium (75 wt %) and indium (25 wt %) to create EGaIn, and we added 14 g of EGaIn to 30 mL of the DMSO:DI water solution. We then used a narrow (3 mm diameter) probe sonicator to add focused acoustic energy to the solution and disperse the bulk LM into droplets.^24^ The remaining oxygen in the solution was still enough to form a thin (1–3 nm) self-passivating Ga_2_O_3_ layer on the EGaIn droplets, which was enough to stabilize the particles and delay coalescence.^25^

### MXene Synthesis

To prepare the MXene solution, we used an HF etching technique from a MAX precursor, Ti_3_AlC_2_. Additional information can be found in the MXene Synthesis Section of the Supporting Information. Once etched and exfoliated using probe sonication, the MXene sheets were on the order of 500 nm–5 μm in diameter and 5 nm thick.^9,26^ The MXenes were suspended in an 80:20 DMSO:DI water solution with 10 wt % Ti_3_C_2_T_*x*_ MXene. This solvent mitigates restacking that would occur from the large surface energy of the MXenes.^27^ The MXenes exhibited a strong negative zeta potential of −52 mV when diluted in DI water, indicating strong stability of the colloidal suspension (Figure S1a).^10^ Next, the MXene solution was added to the vial containing the LM DMSO solution, with the MXene volume adjusted to achieve the desired concentration. Separately, to confirm the electrical performance of the MXenes, we tested the bulk conductivity of the Ti_3_C_2_T_*x*_ MXenes by vacuum-filtering the solution into a film. Using a 4-point probe test, the bulk conductivity was 5.2 × 10^4^ ± 2.0 × 10^3^ S/m.

### MXene-LM Composite Synthesis

To wrap the MXenes around the LM droplets, we probe sonicated the MXene-LM solution a second time to produce an energetic environment to enable wrapping. Separately, 50 μL of the probe sonicated MXene-LM solution was dropcast into 10 mL of DMSO:DI water solution to heavily dilute the composite to evaluate the bonding strength between MXenes and LM. The solution was then planetary mixed at 2000 rpm for 2 min. We then drop-cast and performed SEM of the solution. The MXene-LM coatings were strong enough that virtually all visible MXene remained coated around and between the LM particles, forming small-scale microaggregates on the order of 5–30 μm (Figure S2a,b). This indicated that even under planetary mixing, the MXenes do not separate from the LM.

Next, the MXene-LM solution was vacuum filtered to remove the solvent and leave the wrapped MXene-LM particles in a paste form. We then transferred the material to a mixing cup filled with a silicone oil. A high viscosity silicone oil (60,000 cSt *@* 25 °C) was selected as the matrix material, to decrease the settling and phase separation. The silicone oil that we selected is noncuring, allowing each batch of MXene-LM composite to function as a fluidic paste without the concern of curing or increased viscosity during repeated testing. The composite was then mixed by hand and planetary mixed for three cycles, causing the MXene-LM particles to aggregate. Finally, the sample was placed in an oven to evaporate any remaining solvent. Additional materials and methods information can be found in the Supporting Information Section.

## Results and Discussion

### Particle Characterization

**Figure 2:**
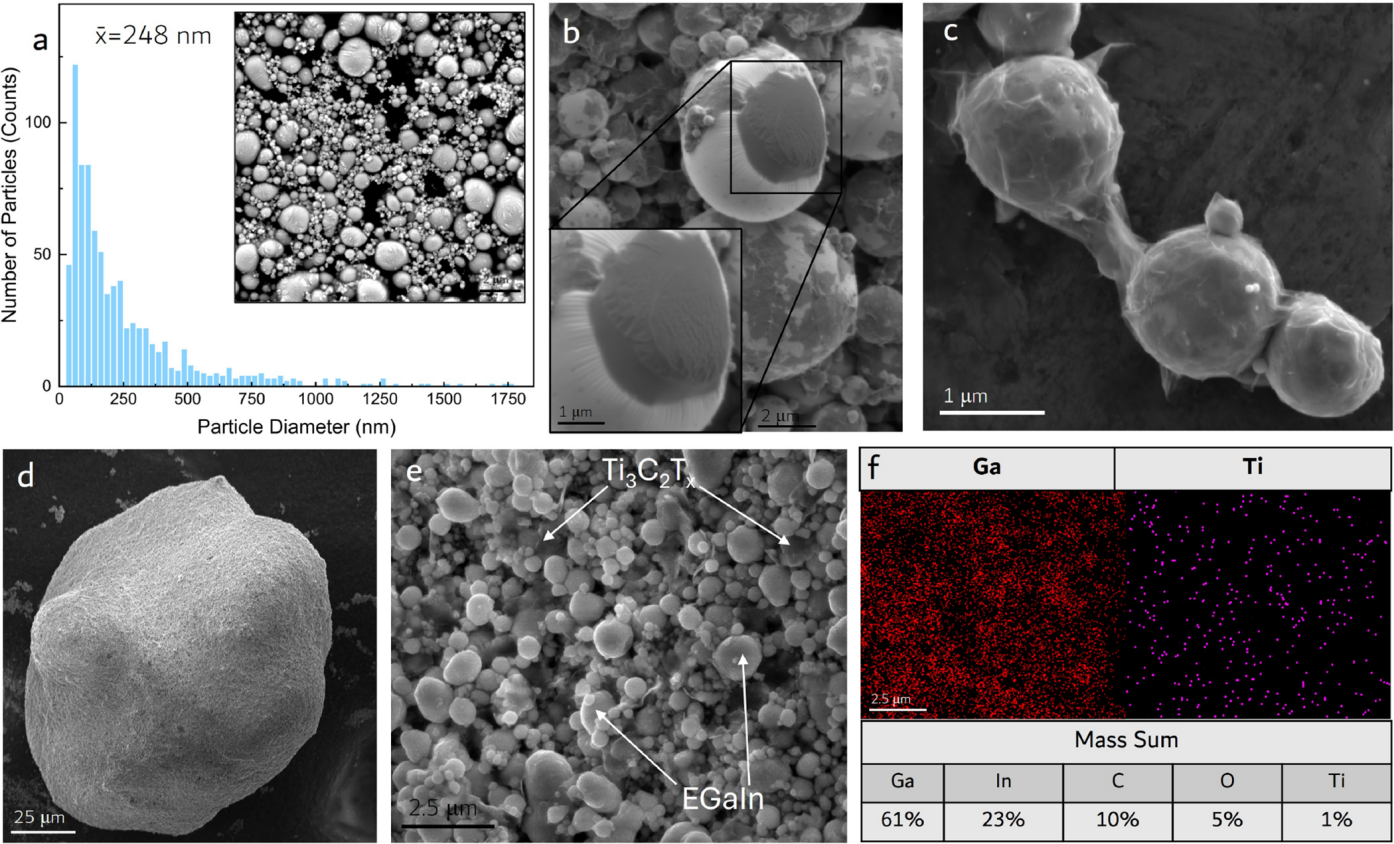
MXene-LM Particle Characteristics: (a) Particle size statistics histogram of a representative sample of LM nanodroplets after probe sonication with an average diameter of 248 nm. Inset: SEM image was used for particle size analysis. (b) SEM image of EGaIn droplets partially coated with Ti_3_C_2_T_*x*_ sheets adhering to the LM surface after processing. Inset: high-magnitude image highlighting the MXene sheets generating tension and wrinkling on the Ga_2_O_3_ skin of the partially coated EGaIn droplet. (c) SEM image of LM droplets being coated and connected with a network of conductive MXene flakes. Image taken from a LM+5.8 vol % MXenes sample. This particular sample was prepared in a lower-oxygen environment. (d) SEM image of a recovered conductive MXene-LM aggregate (LM+5 vol % MXene) formed after planetary mixing in silicone oil. (e) Surface profile of the LM+5 vol % MXene aggregate shown in (d) highlighting intermixed MXene and LM regions. (f) Top: elemental mapping of the aggregate surface in (e) with gallium representing the EGaIn regions, and titanium representing Ti_3_C_2_T_*x*_ regions. Bottom: The total mass sum of all elements present.

Size analysis was conducted on LM particles after a first stage of probe sonication to understand the relative size of the LM droplets used in synthesis. Probe sonication parameters were selected to produce EGaIn particles with an average diameter of 248 nm (Figure 2a). The LM droplets were small enough (Figure 2a inset) that individual MXene flakes (500 nm–5μm diameter) could wrap and coat the droplets, making them conductive and functionalizing their surfaces (Figure 2b,c).

When even a small amount (0.25 vol %) of Ti_3_C_2_T_*x*_ MXenes was added to the LM droplet solution and probe sonicated for the second time, the MXene sheets were observed to wrap around the surface of the EGaIn droplets. Referring to Figure 2b, the MXenes bonded so well to the Ga_2_O_3_ skin that the MXenes induced tension, which caused wrinkling on the Ga_2_O_3_ surface. At this point in fabrication, the Ti_3_C_2_T_*x*_ coating fully wrapped the Ga_2_O_3_ skin of the LM droplets, forming conductive pathways around and between the LM droplets (Figure 2c). As mentioned previously, this MXene wrapping also induced microclustering of the LM droplets, forming 5–30 μm conductive networks. Even after dilution and mixing at 2000 rpm for 2 min, the LM particles were still held together and wrapped by the MXene flakes (Figure S2a,b).

We believe that wrapping occurs primarily due to electrostatic van der Waals interactions. This is hypothesized as MXenes have in the past been shown to act as building blocks to form van der Waals heterostructures when introduced with other 2D nanomaterials.^28^ Recent work has used atomic force microscopy (AFM) methods to demonstrate short interaction ranges of <3 nm between MXenes and SiO_2_ along with graphene, indicating the dominance of van der Waals interactions.^29^ Similarly, 2D self-assembly has been shown between Ti_3_C_2_T_*x*_ onto V_2_O_5_ nanoplates for energy storage.^30^ More specifically, we hypothesize that these van der Waals interactions induce the spontaneous coating of the Ti_3_C_2_T_*x*_ onto the EGaIn as the EGaIn reduces the free surface energy of the Ti_3_C_2_T_*x*_ during sonication.^27^ Experimentally, we observed a large difference in surface charges between MXenes and LM, with a strong negative potential recorded for MXenes and a fairly neutral to weak positive zeta potential recorded for LM indicating some limited evidence of electrostatic interactions (Figure S1a,b).

Due to the 2D nature of MXenes, we discovered that SEM microscopy of individual MXene sheets can oftentimes be difficult, since high accelerating voltages (10 kV) cause the electron beam to penetrate through the surface, giving false surface profiles. One kV SEM microscopy was performed to correct for this. This improved the resolution of the surface morphology helping to highlight the interface of the MXene-LM boundary layer showing uniform and strong adhesion of the MXenes on the Ga_2_O_3_ skin (Figure S3).

### Cluster Aggregation

**Figure 3:**
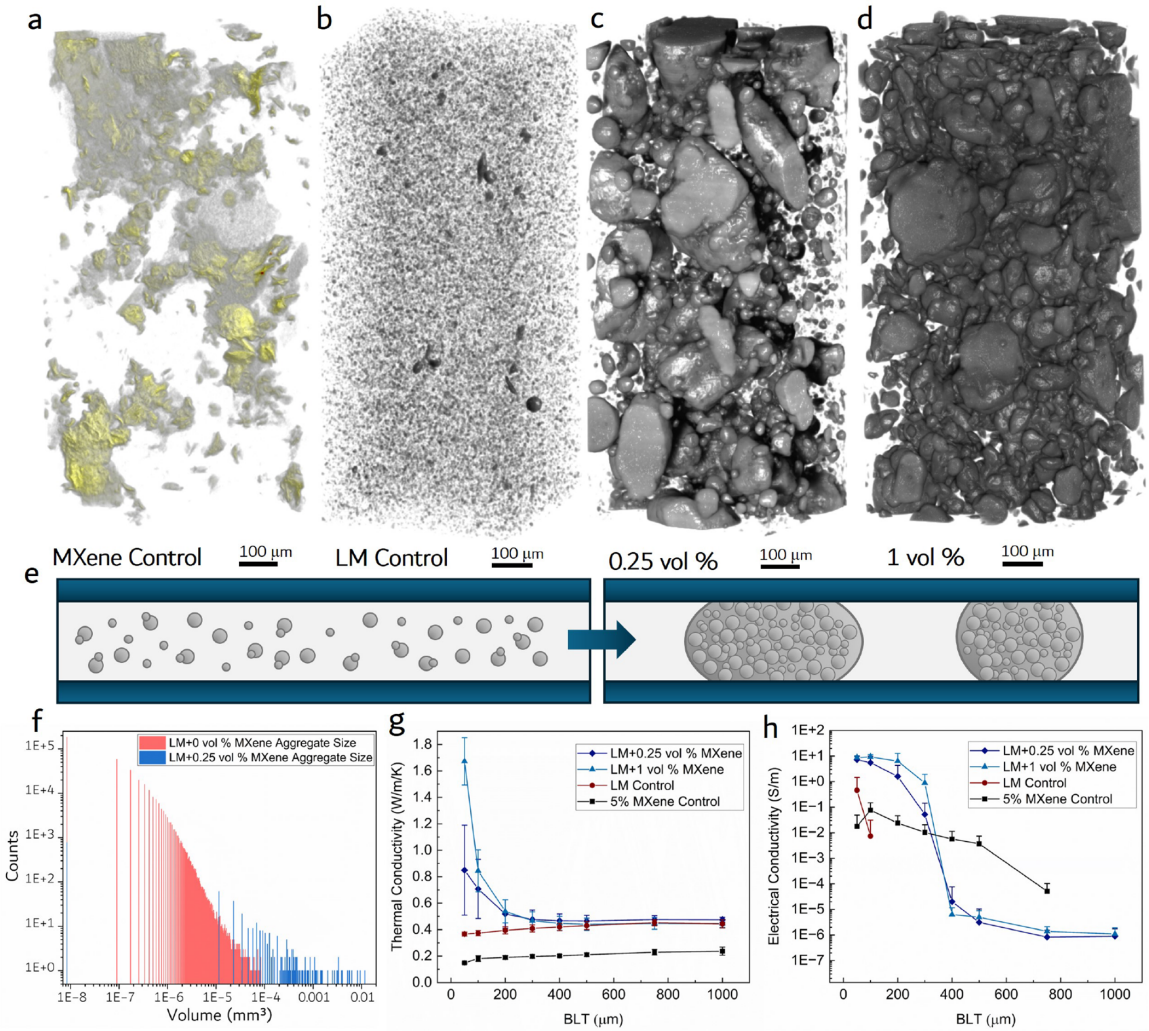
3D MicroCT Reconstructions and Properties: (a) MicroCT 3D reconstruction of a 0 vol % LM+5 vol % MXene control sample with no LM. (b–d) Intensity calibrated grayscaled MicroCT 3D reconstructions of LM control (LM+0 vol % MXene), LM+0.25 vol % MXene, and LM+1 vol % MXene suspended in a silicone oil matrix material. (e) indicating the change in the percolation threshold with and without the self-assembling MXene-LM aggregates between two parallel plates. (f) Histogram of individual cluster sizes for LM+0 vol % MXene control sample and LM+0.25 vol % MXene sample with a 3 order of magnitude average aggregate size increase when 0.25 vol % MXene was added. (g) Thermal conductivity measurements vs BLT for control and MXene-LM samples, highlighting an exponential increase in thermal conductivity as BLT decreases. (h) Electrical conductivity vs BLT for control and MXene-LM samples, indicating a large increase in conductivity with a small volume fraction of MXenes.

Following the characterization of individual particles, silicone oil was added to act as a dispersion medium for the MXene-LM suspension. This was followed by planetary mixing, which caused the sticky microclusters that had formed during sonication to self-assemble into large aggregates when mixed within the viscous silicone oil. MicroCT imaging was conducted to analyze particle aggregation of the MXene-LM composites as the MXene content was varied. Additional details can be found in the Supporting Information. A low MXene filler sample and a high MXene filler sample were prepared along with LM control and MXene control. The MXene control contained 5 vol % MXene suspended in silicone oil, and the LM control contained 25 vol % LM. The low MXene filler sample contained 0.25 vol % MXene as a total volume fraction of the entire composite and 25 vol % LM as a total volume fraction of the entire composite. The high MXene filler sample contained 1 vol % MXene and 25 vol % LM. MicroCT imaging was conducted for each sample (Figure 3a–d). The LM control samples indicated no aggregation (Figure 3e). The LM control sample had an average EGaIn cluster size on the order of 1 × 10^–7^ mm^3^ (Figure 3f). These small droplets and clusters are evenly distributed and are well below the percolation threshold needed for electrically conductive composites (Figure 3b). The microCT reconstructions were intensity calibrated and threshold segmented (see Supporting Information).

Due to the instability of MXenes sheets outside of stable solvents and their tendency to restack and crash out in nonpolar nonaprotic solvents or in environments with non-neutral pHs, the MXene control sample was prepared separately. Without EGaIn particles present for the MXenes sheets to wrap and cling around and help stabilize, it is not possible to remove the solvent without a large film of intercalated MXenes forming as the individual sheets restack and precipitate.^27^ To circumvent this issue, the MXene solution was added to the silicone oil directly during the fabrication process. This solution was then placed on a hot plate and shear mixed, to mitigate the aggregation of the MXenes as the DMSO:H_2_O solution evaporated. The resulting MXene control sample was imaged by using microCT (Figure 3a). The reconstruction instead indicated the formation of large MXene layered sheets throughout the sample. These most likely formed during solvent evaporation. We believe this occurs as the removal of the stable DMSO solution and the addition of the silicone matrix material induced a charge instability on the MXenes in the colloidal solution. This led to a restacking and aggregation of the material along with phase separation in the silicone oil (Figure S4). Images of the crumpled MXene clusters after they have been removed by diluting the sample in toluene to remove the silicone oil are shown in Figure S5.

When a small amount of MXene was added to the LM (0.25 vol % MXene), the composite formed large aggregates dissimilar to the MXene and LM control samples (Figure 3c). See the Supplementary Videos 1, 2, and 3 for comparison animations of the reconstructed microCT tests for the LM control, LM+0.25 vol % MXene, and LM+1 vol % MXene. The aggregates were made of tightly packed micrometer and nanosized MXene-wrapped LM droplets, as shown in Figure 2e. The 3D visualization analysis (Dragonfly, Object Research Systems) conducted on the LM+0.25 vol % MXene aggregate sample (Figure 3c) showed an average cluster size of 10^–4^ mm^3^, a 3 orders of magnitude increase over the LM control (Figure 3f). Volumetric measurements were used as the parameter of comparison instead of the cluster diameters, since the aggregated clusters cannot be assumed to be spherical based on the microCT imaging. We believe the large clusters formed because of the silicone oil viscosity (60,000 cSt) and planetary mixing speed, which combined to generate strong fluidic shear forces. These forces generated enough stress on the droplets to facilitate intimate contact and the formation of dipole–dipole (van der Waals) bonds. As a result, the “sticky” microclusters were pressed together during mixing, forming larger aggregates. When the same MXene-LM composite with 1 vol % MXene sample was prepared in a lower viscosity silicone oil (23,000 cSt), the aggregates were not found to have formed when compared to those synthesized in high viscosity silicone oils (Figure S6). Based on previous work that demonstrated that viscosity plays a role in LM drop size formation during shear,^31^ we suspect that higher speeds and viscosities during mixing will enable enough interfacial stress for van der Waals bonding and aggregate formation.

To analyze the individual aggregate properties, the aggregates themselves were recovered from the silicone oil for analysis. This was done by orbital shaking of the composite material in a toluene bath for 15 h. SEM imaging revealed the surface properties and morphology of the aggregates as shown in Figure 2d. A higher resolution image of the aggregate surface (Figure 2e) highlights the dense EGaIn particles connected by the Ti_3_C_2_T_*x*_. Energy dispersive X-ray spectroscopy (EDS) of this surface (Figure 2f) confirms this, with significant gallium, indium, and oxygen emissions emanating from EGaIn along with carbon, oxygen, and titanium emissions emanating from the intermixed and coated Ti_3_C_2_T_*x*_ sheets (Figure S7).

### Thermal and Electrical Characterization

To examine the influence of MXene-LM aggregation on thermal and electrical properties, we used a thermal interface material analyzer (TIMA) to measure thermal and electrical conductivities as a function of sample thickness. Samples were compressed from an interfacial gap of 1000 to 50 μm while electrical and thermal resistance measurements were simultaneously recorded. The LM control samples’ thermal conductivity remained stable and unaffected as the interfacial bond line thickness (BLT) between the two test heads decreased, with an average of 0.41 ± 0.03 W/m/K (Figure 3g). This is in line with effective medium theory (EMT), which predicts a thermal conductivity of ∼0.40 W/m/K at a 25 vol %.^32^ Thermal conductivity stayed fairly consistent as BLT decreased because the nanoscale EGaIn droplet diameters are 2–3 orders of magnitude below the interfacial BLT of the test. In contrast, for TIMs with large LM inclusions, compression of the BLT below the LM droplet diameter would induce an increase in thermal conductivity, as the bulk properties of the material begin to dominate performance. The 5 vol % MXene control sample indicated similar BLT independent properties with an average thermal conductivity of 0.20 ± 0.03 W/m/K, showing no improvement over bulk silicone oil or elastomers.

In contrast to the control samples, the LM+0.25 vol % MXene and LM+1 vol % MXene samples’ thermal conductivity was observed to increase exponentially as the BLT began to decrease below 300 μm (Figure 3g). Additional data from trials of other MXene filler contents that highlight similar trends can be found in Figure S8) together with the corresponding microCT images shown in Figure S9 A LM+0.1 vol % MXene sample indicated similar thermal results to the LM control but with still some aggregation seen in microCT at this low vol % (Figure S9). For samples with LM+0.25 vol % MXene or more, when the BLT was compressed to distances approaching the diameters of the individual aggregates, the bulk properties of the compressed aggregates began to play a role in the composite performance. This led to a large increase in the thermal conductivity. This increase occurs at ∼300 μm close to the size of the MXene-LM aggregates, as expected. At 50 μm the 1 vol % MXene-LM composite had a thermal conductivity of 1.67 ± 0.18 W/m/K a 4.6× and 11.3× improvement over the LM control and MXene control respectively out performing predictions from effective medium theory for thermal conductivity.^32^ Due to the stochasticity of this material system, we are not making claims on the comparative performance of the MXene fill content but only that the addition of MXene above 0.25 vol % produces an exponential increase in thermal performance at low BLT.

Electrical conductivity indicated a similar trend in terms of the influence of MXene concentration and LM droplet aggregation on the nonlinear relationship between conductivity and BLT. The LM control sample acts as a dielectric and was not electrically conductive until the interfacial BLT was reduced to 100 μm. This electrical conductivity may have been caused by a few larger LM droplets that were not properly probe sonicated during fabrication. The 5 vol % MXene control sample exhibited a less dramatic increase in electrical conductivity with a peak conductivity on the order of 10^–2^ S/m. The LM+0.25 vol % and LM+1 vol % MXene-LM composites again indicated an exponential increase in electrical conductivity generally below 400 μm (Figure 3h). For these samples, electrical conductivity was observed to increase 7 orders of magnitude from ∼10^–6^ to 10^1^ S/m. Again, the stochasticity of these composites made it such that differentiating between the electrical conductivity of samples with varying MXene concentrations was not possible. Additional data can be found in Figure S8.

## Conclusions

In conclusion, this work described a method and investigated the aggregation of EGaIn droplets wrapped with 2D sheets of Ti_3_C_2_T_*x*_ MXenes. These functionalized MXene-LM particles have a sticky surface that enables self-assembly into macroscale aggregates when suspended and mixed within a silicone oil. Depending on the added MXene concentrations, these droplets can form large clusters on the order of a few hundred μm. SEM confirmed the formation of MXene wrapped LM particles, and extensive MicroCT imaging was conducted to compare the microstructure of nonaggregated LM composites with the aggregated MXene wrapped LM composites. Individual clusters were recovered from the matrix material, and EDS was performed to confirm the existence of MXenes and LM in the large aggregates. Lastly, thermal and electrical characterization was conducted, indicating thickness dependent properties of the composites with exponential increases in thermal conductivity and electrical conductivity at low thicknesses. This work demonstrates the first steps in developing a leak-free LM-based TIM composite with significantly lower volume fractions of LM needed to achieve comparable performance. This would bypass the need for high-volume fractions of LM and their subsequent tendency to leak, allowing for wider adoption of LM based soft composite technology for wearables and electrical components.

This work opens a new direction for interfacing 2D MXene materials with nanoscale droplets to enable emergent material properties not previously shown in soft matter systems. Specifically, it shows that MXenes can act as an aggregation mechanism for binding liquid inclusions into a concentrated, semisolid aggregate. This work introduces a new parameter of control, namely, particle aggregation and assembly, when designing soft composite materials. The material properties introduced here demonstrate leak free LM composites with a significant reduction in needed LM volume fraction when compared with nonaggregated composites (Figure 3g). Nonetheless, the methods presented here are still limited by the stochasticity of the nanosynthesis process. In particular, these results indicate a binary relationship between aggregation and nonaggregation depending on the choice of dispersion medium and processing conditions. Further effort is required to better understand the processing conditions that influence aggregation and to develop more robust synthesis techniques. Future work should also focus on additional experimental studies to better understand how the concentration of MXene can influence the aggregate size, along with developing these MXene-LM composites for use in a cured silicone elastomer for applications in stretchable electronics. In addition, it would be helpful to gain further insight into the role of LM droplet size on aggregate formation and resistance to rupture and leakage for use as a TIM in semiconductor packaging applications.

## Supporting Information Available

Methods, SEM and EDS imaging, microCT reconstructions, and additional thermal and electrical data (PDF)
LM control microCT reconstruction (MPG)
LM+0.25 vol % MXene microCT reconstruction (MPG)
LM+1 vol % MXene microCT reconstruction (MPG)

## Supplementary Material

la5c00173_si_001.pdf

la5c00173_si_002.mpg

la5c00173_si_003.mpg

la5c00173_si_004.mpg
