# Supplementary material for: MXene-Coated Liquid Metal Nanodroplet Aggregates
Source: Langmuir. 2025 Mar 26;41(13):8834–41. doi: 10.1021/acs.langmuir.5c00173 (PMC11984108; doi:10.1021/acs.langmuir.5c00173)
Supplement: Supplementary file 1 — la5c00173_si_001.pdf [file la5c00173_si_001.pdf]

Supporting Information: MXene-Coated Liquid Metal Nanodroplet Aggregates

Mason Zadan,[†] Yafeng Hu,[†] Jeremiah Lipp,[‡] Michael Vinciguerra,[†] Neal Lewis,[¶] Dylan Shah,[§] Mohammad F. Islam,[¶] Dhriti Nepal,[‡] Matthew Grasinger,[‡] Kaushik Dayal,[‖] Christopher Tabor,[‡] and Carmel Majidi[*,†]

†*Mechanical Engineering Department, Carnegie Mellon University, Pittsburgh, PA, 15213, USA*

‡*Materials and Manufacturing Directorate, Air Force Research Laboratory, Dayton, OH, 45433, USA*

¶*Materials Science and Engineering Department, Carnegie Mellon University, Pittsburgh, PA, 15213, USA*

§*Arieca Inc., Pittsburgh, PA, 15208, USA*

‖*Civil and Environmental Engineering Department, Carnegie Mellon University, Pittsburgh, PA, 15213, USA*

E-mail: cmajidi@andrew.cmu.edu

Table of Contents

Content	Page
Supporting Videos	S3
Detailed Methods	S3
MXene Synthesis	S3
Fabrication Steps	S3
MXene Control Composite Synthesis	S5
Thermal and Electrical Characterization	S5
MicroCT Imaging	S5
MicroCT Data Processing	S6
Zeta Potential Testing	S6
Data Processing	S7
Supporting Figures	S7
Figure S1: Zeta potential measurements of MXenes and LM	S7
Figure S2: SEM images of MXene-LM microclusters	S8
Figure S3: Surface profile of MXene coated LM droplets	S8
Figure S4: Extruded LM-MXene composite materials	S9
Figure S5: Images of crumpled and restacked MXene aggregates	S9
Figure S6: High vs low viscosity composite microCT comparison	S10
Figure S7: EDS elemental analysis	S11
Figure S8: Thermal and electrical conductivity data	S11
Figure S9: Additional microCT reconstructions	S12

Supporting Videos

Video 1: LM control microCT reconstruction

Video 2: LM+0.25 vol % MXene microCT reconstruction

Video 3: LM+1 vol % MXene microCT reconstruction

Detailed Methods

MXene Synthesis

MXene preparation was adapted from Shuck et al.[1] The preparation of pristine $Ti_3C_2T_x$ involved the selective etching of the chemical bonds between transition metals and aluminum elements in the Ti_3AlC_2 MAX phase. The first key step is the in-situ generation of HF with highly concentrated LiF and HCl, which selectively removes the Al layer, resulting in accordion-like two-dimensional layered structures. Subsequent intercalation in DMSO with probe sonication and washing steps resulted in 8-10 wt% of exfoliated $Ti_3C_2T_x$ sheets in a colloidal dispersion of 80:20 DMSO: H_2O. The termination layers (T_x) were a mix of -OH, -O, and -F.

Fabrication Steps

MXene-LM composite synthesis began with preparation of the EGaIn LM (75 wt % Ga and 25 wt % In). Ga and In ingots (Rotometals, 99.99 % purity) were alloyed at 180°C on a hotplate overnight and periodically mixed by hand. For processing, a colloidal suspension of 80:20 DMSO (dimethyl sulfoxide, Sigma-Aldrich $\geq$ 99.99%): H_2O was prepared. 30 mL of the DMSO: H_2O solution was added to a 60 mL glass vial with a 30 mm base diameter. This solution was then degassed for 6 minutes in a desiccator using a vacuum pump. 14 g of EGaIn was then added to the solution. Next the LM was processed using a probe sonicator (Sonics, VCX 500). The EGaIn was probe sonicated for 1 hour using using a 3.2 mm diameter

microtip probe (Qsonica, 418A 1/8") placed approximately 1 cm above the base of the vial in an ice bath. The probe sonicator parameters were, 20 kHz, 500 W, 40 % amplitude, with a 8 s on and 1 s off cycle time. For the LM control sample, the contents were then vacuum filtrated. For samples with MXenes added, the correct amount of MXene solution was added to the vial. For example, for a LM+1 vol % MXene sample, 2.715 g (0.674g MXene solution for the LM+0.25 vol % sample) $Ti_3C_2T_x$ solution was added. This was then hand mixed for 15 s. The solution was then probe sonicated again this time using a larger 6.4 mm diameter microtip probe (Qsonica, 420A 1/4") at 20 % amplitude for 30 minutes in an ice bath. The solution was then removed and vacuum filtrated using a 200 nm pore size filter (Nalgene vacuum filtration system) until the solvent was removed and the remaining material resembled a paste like consistency. For select samples, the fabrication process was performed in a low oxygen environment to see if this visually affected the wrapping (210 ppm O_2). This process was identical, but it started in glove box with a nitrogen purge of the MXene solution in DMSO:DI water followed by the addition of bulk LM. The MXenes and LM were then probe sonicated together for 5 minutes in the low O_2 environment with only one short sonication step instead of two (Figure 2c).

The remaining material was then added to a planetary mixing cup and 6.586 g silicone oil was added (Dow Corning Corporation 200 ® fluid, 60,000 cSt @ 25 $°C$). For the low viscosity sample, 7.207 g silicone oil were added (Smooth-On Dragon Skin ™10 Slow Part A, 23,000 cSt). The composite material was then hand mixed for 1 minute. The material was then planetary mixed (Thinky, AR-100 conditioning mixer) for 5 minutes at 2000 rpm. This mixing process was then repeated 2 more times. Lastly, the composite was placed in an oven at 60 $°C$ overnight to evaporate any remaining DMSO:H_2O solution and loaded into a syringe.

MXene Control Composite Synthesis

7.074g MXene solution and 4.390g silicone oil were added to a mixing cup. This solution was placed on a hot plate at 170°C. An overhead shear mixer was then placed into the solution with a 24 mm diameter mixing head (Scilogex , OS20-SO). The solution was then mixed for 3 hours at 200 rpm. This was followed by being placed in an oven at 60°C overnight to remove any additional solvent.

Thermal and Electrical Characterization

For thermal testing, a TIMA was used (TIMA5, Nanotest) to record the thermal conductivity of MXene-LM composites at various thicknesses from 50-1000 μm. The heated head was set to 50 °C and the cooled head chilled to 15 °C. Electrical conductivity measurements were conducted in parallel with multimeter leads connected to the test heads (Keithley 2100 6 1/2 digit multimeter). For the electrical and thermal data, samples divided by MXene filler content were averaged and graphed. Error bars are the standard deviation with only the positive error being shown on the log scale electrical conductivity graph.

MicroCT Imaging

MicroCT Imaging was conducted using a Zeiss Xradia CrystalCT. Samples for imaging were prepared by extruding material into a 10 μL auto-pipette tip. The tip was then cut with the top section being mounted to the chuck using Bondic UV curable resin. The sample was left to settle for a few hours before imaging to limit the amount of drift in the silicone oil suspension. Depending on x-ray beam intensity opaqueness of the sample during setup, the voltage and power were set to either 70 kV at 6 W or 60 kV at 5 W. Pixel resolution was $\sim 1.4 \mu m$. Imaging was conducted with 3600 projections and then manually reconstructed using Scout and Scan (Zeiss) by adjusting for center shift and beam hardening when needed. Separately, SEM imaging was conducted using the FEI Quanta 600 and EDS measurements

were taken using the Tescan Mira 3.

MicroCT Data Processing

Comet Dragonfly software was used for all image analysis following 3D reconstruction. The LM+0.25 vol % MXene sample was used as the calibration control, with all other samples (aside from the MXene control) intensity calibrated to this sample. For intensity calibration, a representative area was averaged of the plastic pipette tube and the LM based clusters; these average values were used as the intensity calibration material, allowing for linear fitting. An upper Otsu image thresholding method was used on the LM+0.25 vol % MXene sample with this calculated intensity threshold then being used on all other intensity calibrated samples. Volumes of $500 \times 500 \times 1200$ μm were cropped for images and videos used here.

For determining the particle size distribution of the LM+0 vol % MXene control sample, an upper Otsu threshold was applied to the intensity calibrated sample as before. The sample was segmented leaving only particles above the Otsu threshold. A multiple region of interest (ROI) was generated from the segmentation. Regions of high density with multiple particles in contact acting as size outliers were manually disconnected by deleting connecting voxels. The process was repeated for the LM+0.25 vol % MXene sample. Due to the large aggregates formed, multiple aggregates were in contact with each other and not able to be differentiated during segmentation. Voxel erosion was used to shrink the particles and separate them using a cross-shaped 3D kernel with a size of 3 for 10 erosion cycles. Once eroded, additional aggregate outliers were manually separated as before. For both samples a histogram was then generated to determine mean particle size. For both samples the binning was set to 1024.

Zeta Potential Testing

LM and MXene zeta potential were measured in a DTS1070 folded capillary zeta cell by a Malvern Zetasizer Nano ZS particle size analyzer. $10\,\mu L$ of the LM and MXene suspension

in DMSO:DI water was diluted with $10\,mL$ of distilled water before zeta-potential measurements.

Data Processing

We perform 2D image particle size analysis on SEM images using ImageJ software. The image was thresholded to create a binary image, where the filler appears as bright areas and the matrix as dark areas. A built-in particle analysis is utilized to calculate these separated areas. The radius is calculated assuming circular areas and is plotted as histograms.

Supporting Figures

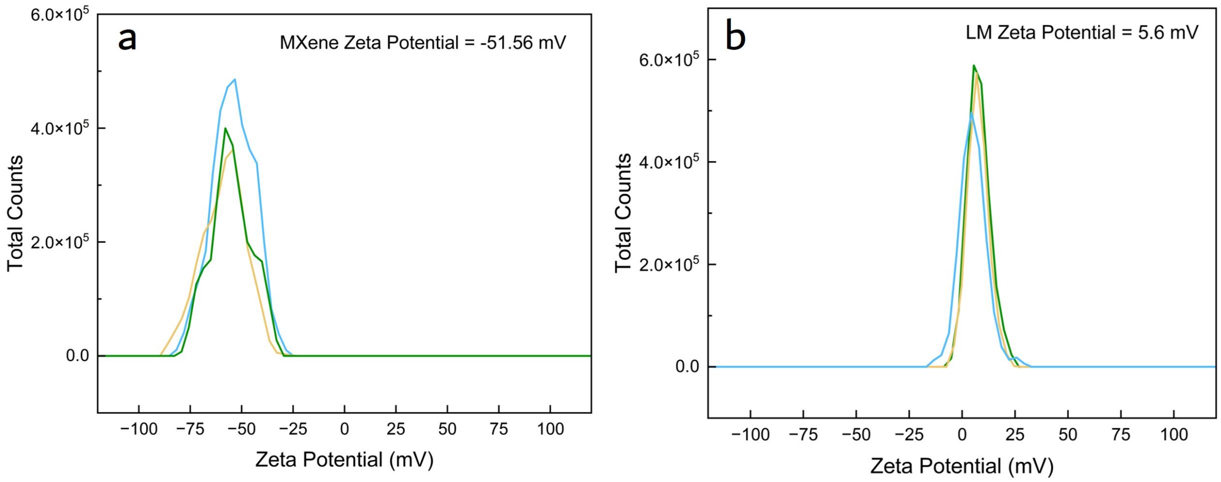

Figure S1: **a**, Three cycles of zeta potential measurements for MXenes prepared in 80:20 DMSO:DI Water and diluted in DI water indicating strong negative charge and colloidal stability. **b**, Three cycles of zeta potential measurements for LM prepared in 80:20 DMSO:DI Water and diluted in DI water indicating weak surface charges and poor colloidal stability. It should be noted that zeta potentials will vary depending on the solvent diluted in, making these results not generalizable and relevant for the particular solvents in question.

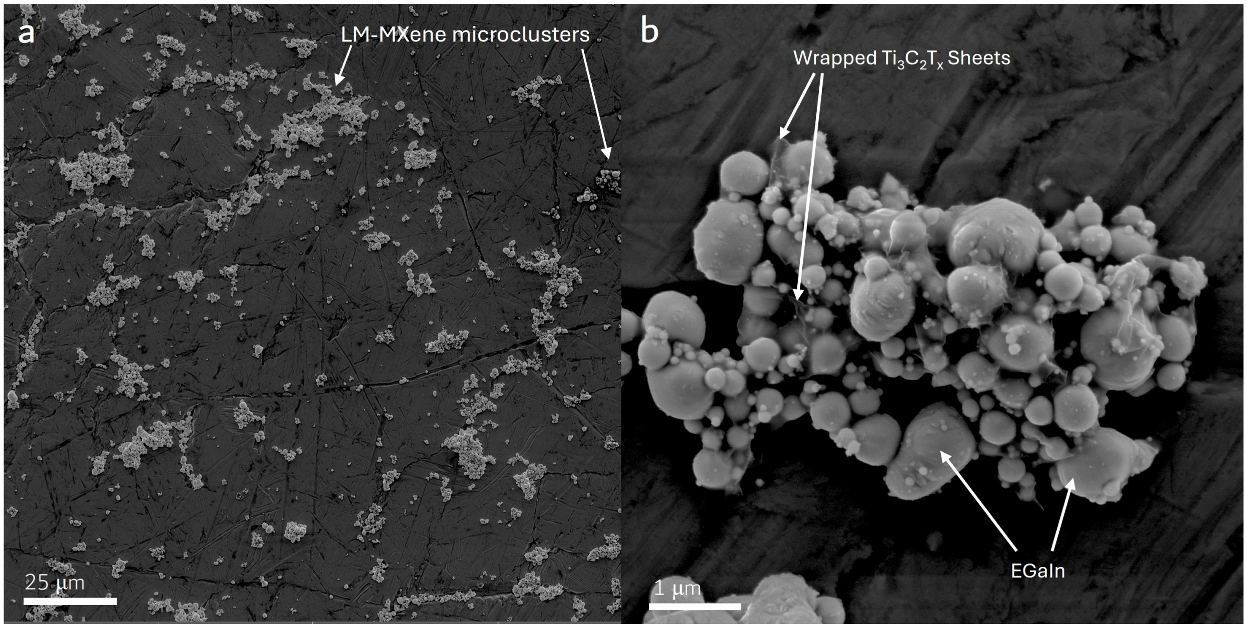

Figure S2: **a**, SEM Image of MXene-LM microclusters on an Al stub after sonication of MXenes and LM in a DMSO:DI water solution. After sonication, the solution was then diluted and planetary mixed before drop-casting onto the Al stub. **b**, Image of one individual microcluster highlighting the MXene sheets wrapping and binding the LM droplets together.

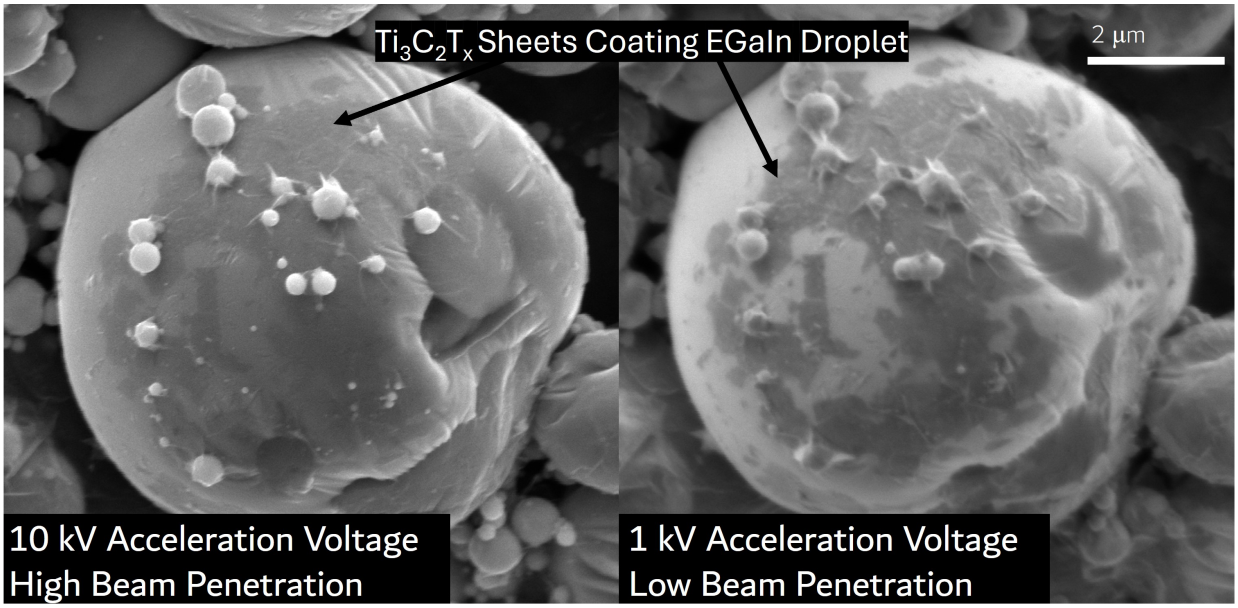

Figure S3: Image of a large LM droplet and smaller LM droplets on the surface coated with MXene sheets after probe sonication. This figure gives insight into the influence of SEM accelerating voltage on beam penetration and the quality of 2D MXene sheet images.

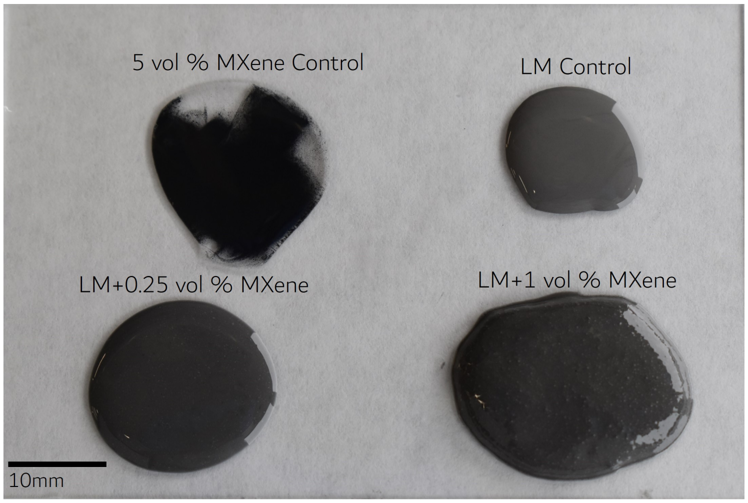

Figure S4: Images of fabricated LM-MXene composite material in high viscosity silicone oil with apparent phase separation in the MXene control sample and visible aggregate formation in the LM+1 vol % MXene sample.

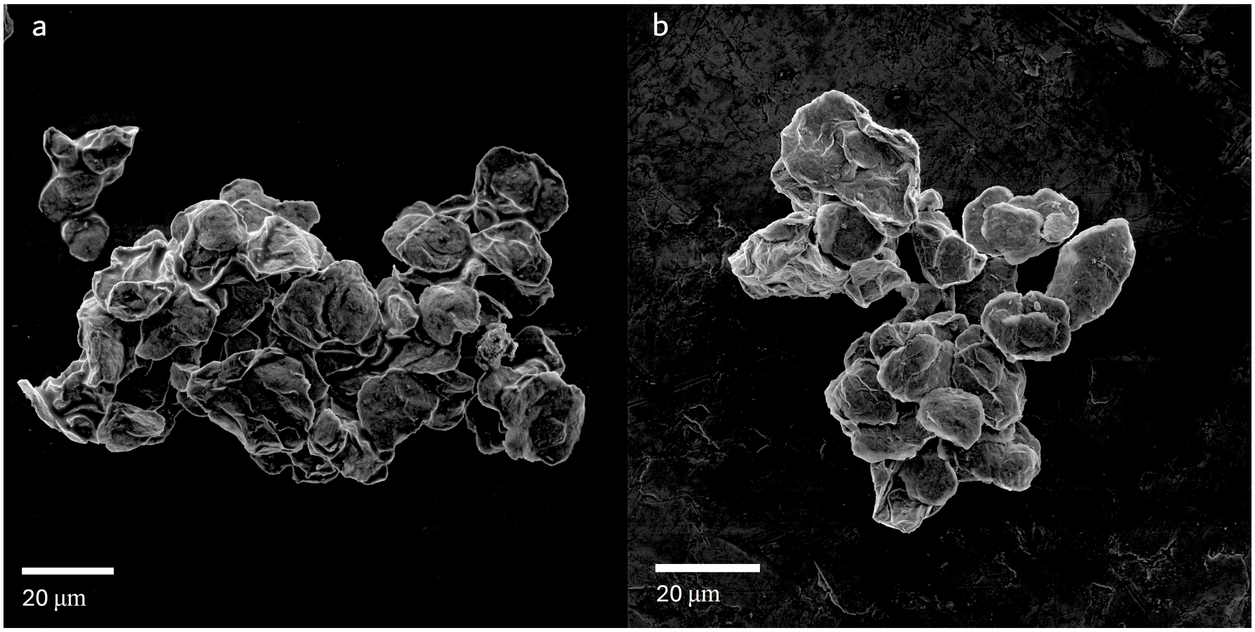

Figure S5: **a-b**, Images of crumpled and restacked MXene aggregates recovered from silicone matrix material. Images are taken from a 5 vol % MXene control sample.

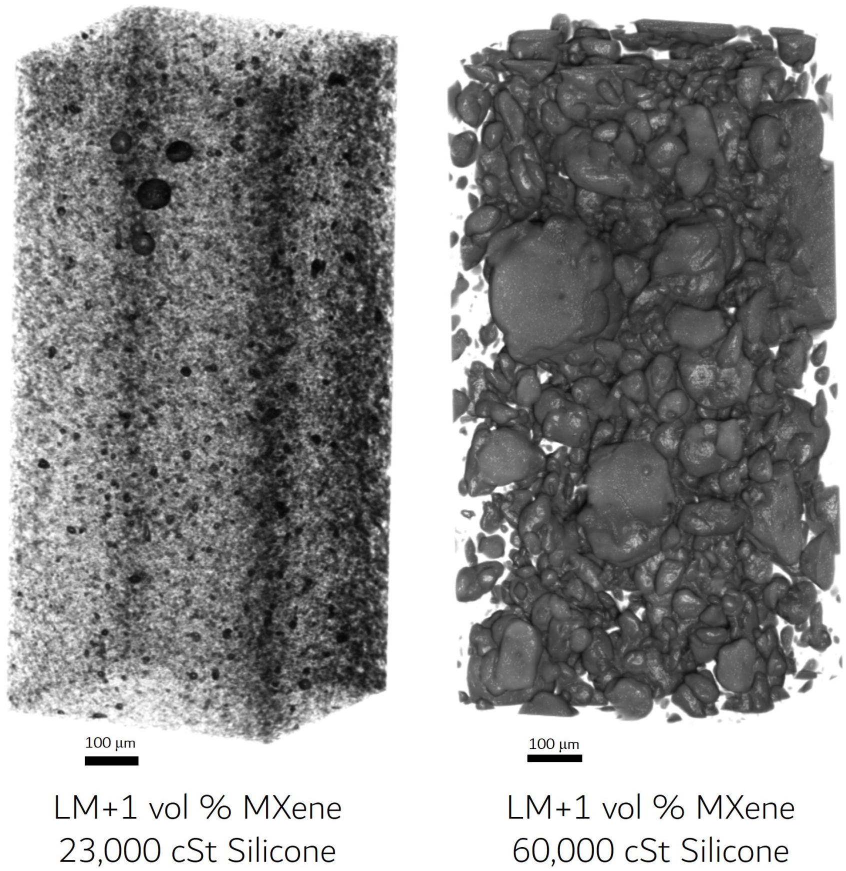

Figure S6: Intensity calibrated MicroCT reconstructions of LM+1 vol % MXene samples prepared in 23,000 cSt silicone (left) and 60,000 cSt silicone (right). Larger aggregates were formed in the 60,000 cSt silicone sample, indicating that friction is causing the aggregation.

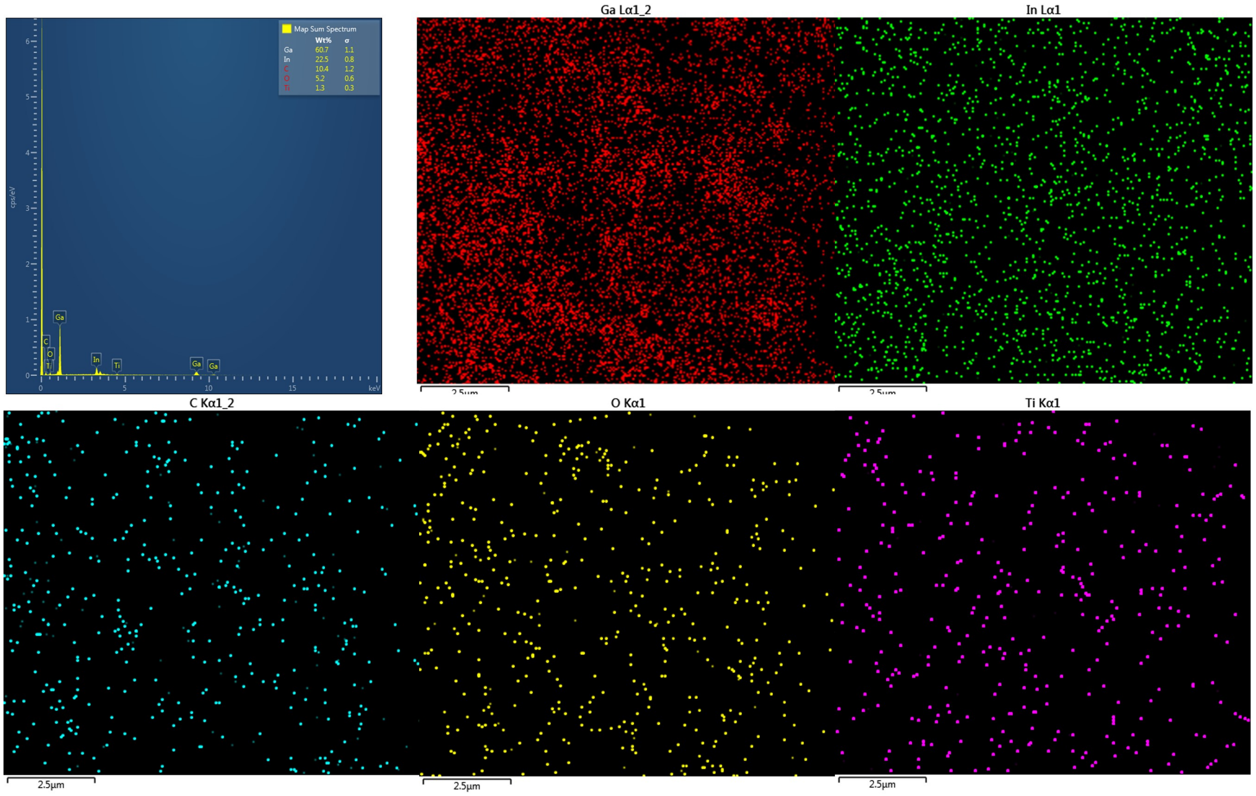

Figure S7: EDS map sum spectrum corresponding to Figure 2e, along with individual elemental maps.

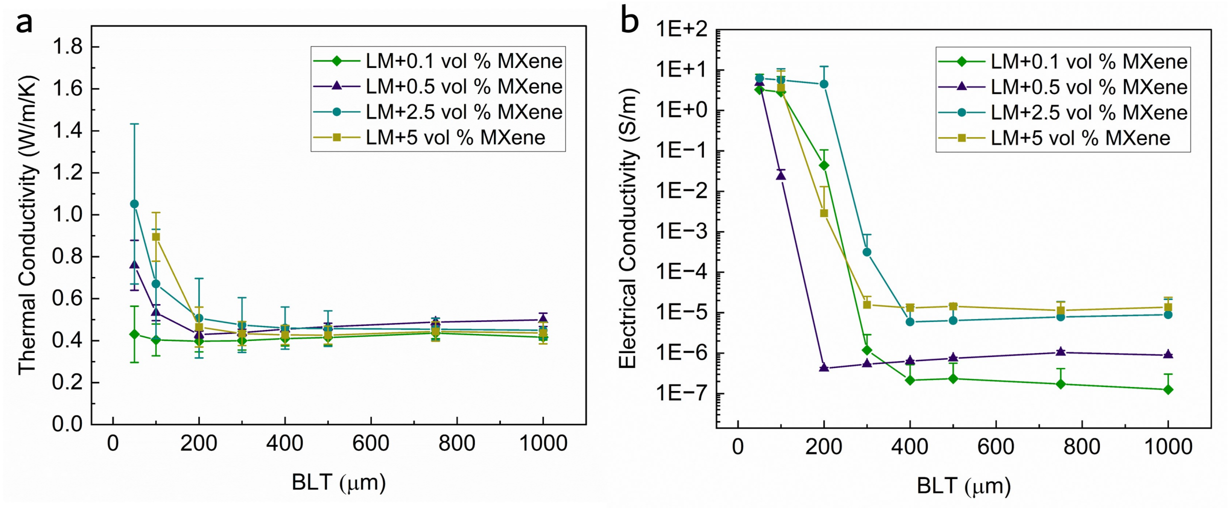

Figure S8: **a**, Graph of thermal conductivity vs. BLT for various MXene vol %. **b**, Graph of electrical conductivity vs. BLT for various MXene vol %.

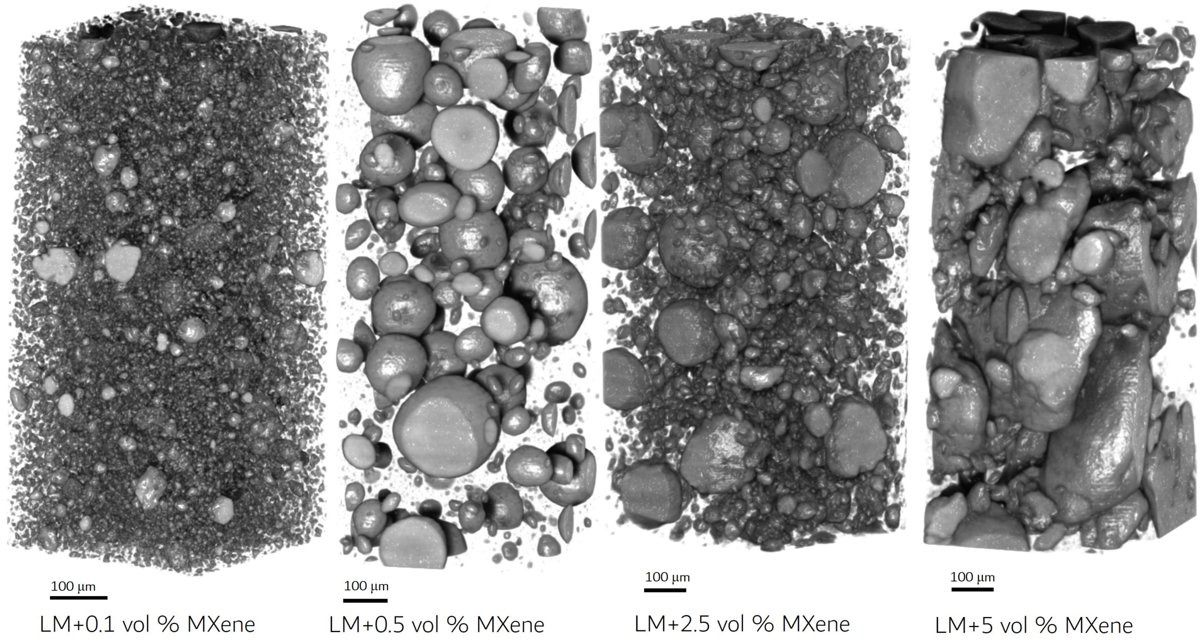

Figure S9: Intensity calibrated microCT reconstructions of MXene-LM composites for additional volume fractions. Aggregation is shown with as little as LM+0.1 vol % MXenes when compared with the LM control (Figure 3b).

References

(1) Shuck, C. E.; Sarycheva, A.; Anayee, M.; Levitt, A.; Zhu, Y.; Uzun, S.; Balitskiy, V.; Zahorodna, V.; Gogotsi, O.; Gogotsi, Y. Scalable synthesis of Ti3C2Tx mxene. *Adv. Eng. Mater.* **2020**, *22*, 1901241.
